# Apoptosis and telomeres shortening related to HIV-1 induced oxidative stress in an astrocytoma cell line

**DOI:** 10.1186/1471-2202-10-51

**Published:** 2009-05-22

**Authors:** Michela Pollicita, Carolina Muscoli, Antonella Sgura, Alberto Biasin, Teresa Granato, Laura Masuelli, Vincenzo Mollace, Caterina Tanzarella, Claudio Del Duca, Paola Rodinò, Carlo Federico Perno, Stefano Aquaro

**Affiliations:** 1 Department of Experimental Medicine and Biochemical Sciences, University Tor Vergata, Rome, Italy; 2 Faculty of Pharmacy, University Magna Graecia, Catanzaro, Italy; 3 IRCCS, San Raffaele La Pisana, Rome, Italy; 4 Pharmaceutical Biotecnogical Center, University Tor Vergata, Rome, Italy; 5 Department Biology, University Roma3, Rome, Italy; 6 Institute of Molecular Biology and Pathology IBPM-CNR, Rome, Italy; 7 Department of Experimental Medicine, University Sapienza, Rome, Italy; 8 CETA, ARPACal, Catanzaro, Italy; 9 Department of Biology, University Tor Vergata, Rome, Italy; 10 Department of Pharmaco-Biology, University of Calabria, Rende, Italy

## Abstract

**Background:**

Oxidative stress plays a key role in the neuropathogenesis of Human Immunodeficiency Virus-1 (HIV-1) infection causing apoptosis of astroglia cells and neurons. Recent data have shown that oxidative stress is also responsible for the acceleration of human fibroblast telomere shortening in vitro. In the present study we analyzed the potential relations occurring between free radicals formation and telomere length during HIV-1 mediated astroglial death.

**Results:**

To this end, U373 human astrocytoma cells have been directly exposed to X4-using HIV-1IIIB strain, for 1, 3 or 5 days and treated (where requested) with N-acetylcysteine (NAC), a cysteine donor involved in the synthesis of glutathione (GSH, a cellular antioxidant) and apoptosis has been evaluated by FACS analysis. Quantitative-FISH (Q-FISH) has been employed for studying the telomere length while intracellular reduced/oxidized glutathione (GSH/GSSG) ratio has been determined by High-Performance Liquid Chromatography (HPLC). Incubation of U373 with HIV-1IIIB led to significant induction of cellular apoptosis that was reduced in the presence of 1 mM NAC. Moreover, NAC improved the GSH/GSSG, a sensitive indicator of oxidative stress, that significantly decreased after HIV-1IIIB exposure in U373. Analysis of telomere length in HIV-1 exposed U373 showed a statistically significant telomere shortening, that was completely reverted in NAC-treated U373.

**Conclusion:**

Our results support the role of HIV-1-mediated oxidative stress in astrocytic death and the importance of antioxidant compounds in preventing these cellular damages. Moreover, these data indicate that the telomere structure, target for oxidative damage, could be the key sensor of cell apoptosis induced by oxidative stress after HIV infection.

## Background

Oxidative stress has been shown to contribute to apoptotic cell death occurring in AIDS-dementia complex (ADC) [1]. Despite the demonstrated role of free radicals in ADC, the mechanism underlying HIV related oxidative damage of central nervous system (CNS) is still unknown. HIV-1 proteins such as gp120 and Tat can cause free radical production, possibly as part of their signal-transduction pathways activation [2]. It has previously been shown that gp120 can cause lipid peroxidation and production of hydroxynonenal esters [3] which in turn can mediate oxidative stress induced apoptosis of cultured neurons and cause cognitive dysfunction in vivo [4]. Significantly, greater numbers of apoptotic astrocytes were detected in the brain of HIV/AIDS patients with rapidly progressing dementia [5], and detection of apoptotic astrocytes appeared to be more common in patients with dementia, compared to non-demented HIV/AIDS patients [6], suggesting a role for astrocytic cell loss in the neuropathogenesis of HIV-1 associated dementia (HAD). It has been demonstrated that incubation of human cultured astroglial cells with the supernatants of HIV-1 infected monocytes derived macrophages (MDM) leads to apoptotic cell death of astrocytes (not infected and not necessary adjacent to HIV-infected MDM), an effect that is driven by overproduction of superoxide anions [7]. Oxidative stress contributes to many aspects of HIV-1 disease pathogenesis, including viral replication, inflammatory response, decreased immune-cell proliferation and loss of immune function [7]; moreover, it leads to the production of reactive oxygen species that can attack lipid membranes, proteins, and deoxynucleic acids resulting in cellular dysfunction and cell death [8]. Moreover, cellular oxidative stress levels directly and quantitatively determine the rate of telomere shortening [9]. Telomeres are heterochromatin regions at the end of linear chromosomes, composed of a double-stranded region (of several Kbp.) and of a single stranded extremity (150–300 bases), responsible for chromosome stability and cell viability [10]. More recently, experimental evidence has accrued that addresses the challenging question of if and how telomere length regulation may contribute to normal human aging or to human disease [11,12]. The presence of telomeres, constituted by short, tandem DNA repeats of the 5'-(TTAGGG)n-3'sequence and a multitude of associated proteins, distinguishes the natural ends of chromosomes from random DNA breaks, thereby preventing unwanted end-to-end fusion or nucleolytic degradation [13-15]. A dysfunctional telomere is detected as damaged DNA and results in activation of the DNA-damage checkpoint, and increased apoptosis [10]. Apoptotic loss of progenitor cells in response to telomere shortening stimuli has been clearly demonstrated in animal models; e.g., mice with shortened dysfunctional telomeres demonstrate increased apoptosis in germ cells of the testes and crypt cells of the intestine [16,17]. In these systems, an increase in apoptosis correlates with tissue atrophy and other phenotypes associated with premature aging. The principal protein involved in telomere maintenance in human cells is the ribonucleoprotein enzyme telomerase, that adds the repetitive sequences to the ends of chromosomes, thus compensating for the end replication problem and thus stabilizes the lengths of telomeres, allowing cells to divide indefinitely [18]. In general, somatic cells do not express telomerase and their replicative potential is limited by progressive telomere shortening, eventually resulting in the onset of cellular senescence. In contrast, cells that constitutively express telomerase are able to divide almost indefinitely [19]. In vitro infection of human PBMC with HIV-1 down-modulates telomerase activity [20] that is down-regulated at both nuclear and cytoplasmic compartments [21]. Oxidative stress is responsible for telomere shortening accelerations in human fibroblast *in vitro *[22-26]. Free radicals enhance induction of telomeric single strand breaks leading to the loss of the distal fragments of telomeric DNA following replication [27]. Further studies have shown that telomeric DNA is a preferential target for oxidative damage [28-30] and accelerated telomere shortening has been detected in cells from patients with mutations in mitochondrial DNA characterized by an increased production of reactive oxygen species [31] but the relative contributions of these different mechanisms to telomere shortening still remain unknown, although oxidative stress has been suggested as one of the major causes of telomere shortening [24]. The relationship among neuroAIDS/oxidative stress and oxidative stress/telomere and telomerase modulation is an important issue for better understand the role of the ends of eukaryotic chromosomes and telomerase activity regulation in HIV-related neuroimmune disorders. The purpose of our work was to evaluate the link existing between HIV-1-induced oxidative stress and cellular damage, such as apoptosis, alterations of telomeric structures and glutathione (GSH/GSSG) redox system in a human astrocytoma cell line.

## Results

### HIV-1 mediated apoptosis in human astrocytoma cell line

**Figure 1:**
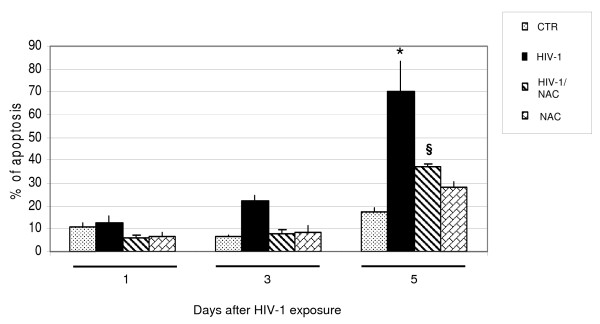
**Percentage of apoptosis in HIV-1 IIIB exposed U373 (8,000 pg/ml) and/or NAC treated compared to negative control after different times (day 1; day 3; day 5)**. The apoptotic cells were stained with propidium iodide (PI) and analysed by flow cytometry. The percentage of apoptotic cells was significantly lower for infected NAC-treated vs HIV treated cells. *P < 0.01 when compared vs control; §P < 0.01 when compared vs HIV-1. F: 5.84, 23.63, 32.42, respectively at day 1, 3, 5.

Apoptosis has been evaluated and statistically analyzed in HIV-1 exposed U373, NAC treated and HIV-1-exposed U373, in mock HIV-infected and not NAC treated U373 (negative control) and in NAC treated U373 at day 1, day 3, day 5 post exposure. We chose the days 1, 3 and 5 because in these days it is possible to observe an increment of apoptosis; after the day 5 we can observe only a plateau. HIV-p24 antigen production in supernatants of HIV-1 exposed astrocytes was negative (data not shown. ELISA kit HIV-p24 gag, Biorad, Italy). A significant induction of apoptosis was seen in a time dependent fashion with a maximum of 70.2% at day 5 post exposure in HIV-1 IIIB exposed U373. In U373 exposed to HIV-1 Bal the value of apoptosis at day 5 is only 22.5%; so the increment of apoptosis at day 5 for HIV-1 IIIB is around 80%, whereas for HIV-1 Bal is around 21%. For this reason in our experiments we used the HIV-1 IIIB. Apoptosis in HIV-1 exposed samples observed at day 3 and 5 was three and four fold higher compared to negative control, respectively (22.1% compared to 7% at day 3, and 70.2% compared to 17.8 at day 5; Figure 1). HIV-1 mediated apoptosis was reduced in U373 by treatment with the antioxidant compound NAC (1 mM), (71.1% and 47.1% of apoptosis reduction after 3 and 5 days of HIV-1 virus exposure respectively, Figure 1); this result underlines the role of oxidative stress in the apoptotic process. Viability test by using trypan blue, at days 1, 3 and 5, revealed that the NAC treatment is not toxic for the cells.

**Figure 2:**
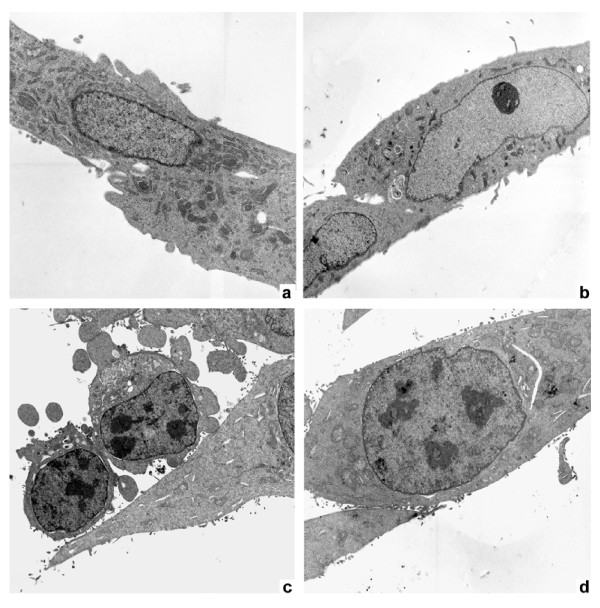
**Ultrastructural analysis of astroglial cells**. (a) Control cell line. The cells are large with irregular nuclei composed mainly by euchromatin with a few peripheric heterochromatin. Numerous dense mitochondria, endoplasmic reticulum are shown in the cytoplasm (original magnification, ×3800). (b) Incubation of astroglial cells with NAC did not modify ultrastructural images of astroglial cells (original magnification, ×3800). (c) Astroglial cells exposed to HIV-1 undergo apoptotic cell death 3 days after exposure. In fact, the cells displayed an increase of plasma-membrane protrusions and cytoplasmic blabbing and large vacuoles as a result of cytoplasmic loss. The chromatin is condensed and marginalized, expressing DNA fragmentation (original magnification, ×3800). (d) The effect of HIV on astroglial cells is strongly antagonized by coincubation with NAC. In particular, it is shown that cells maintain the normal architecture and the normal ratio between cytoplasm and nuclei, which appear almost completely normal (original magnification, ×3800).

These results were confirmed by ultrastructural studies with electron microscopy. At day 3 after exposure to HIV-1 IIIB, astrocytes showed an increase of plasmamembrane protrusions and in many cells, a developed cytoplasmic blabbing and large vacuoles as a result of cytoplasmic loss (Figure 2c) as compared to the control cell line (Figure 2a). Moreover the chromatin was seen condensed and marginalized (Figure 2c). The effect of HIV on astroglial cells was antagonized strongly by coincubation with NAC (1 mM). In particular, we found that in NAC-pretreated astrocytes, cells maintained the normal architecture and the normal ratio between nucleus and cytoplasm, and nuclei appeared almost completely normal (Figure 2d). The treatment with NAC on control cell line did not induce any change (Figure 2b).

### HIV-1 exposure induces the telomeres shortening in human astrocytoma cell line

**Figure 3:**
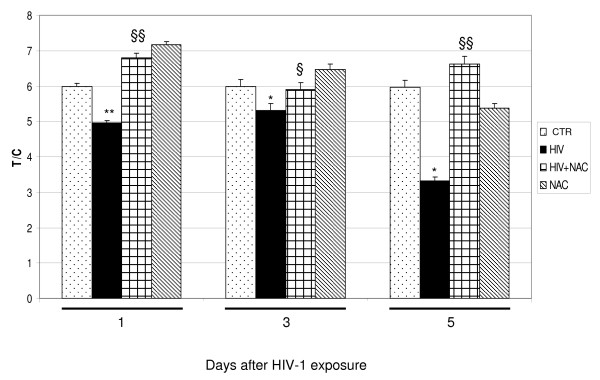
**Telomere length in U373 at different time points (1, 3, 5 days) following HIV-1 exposure or NAC treatment**. Each data is shown as percentage of T/C (telomere/centromere) ratio. A statistically significant telomere shortening was observed for U373 after HIV-1 exposition at all time points while NAC treatment was able to inhibit this effect. *P < 0.05 and **P < 0.001 when compared vs control; §P < 0.05 and §§ P < 0.001 when compared vs HIV-1. F: 96.59, 9.01, 8229, respectively at day 1, 3, 5.

**Figure 4:**
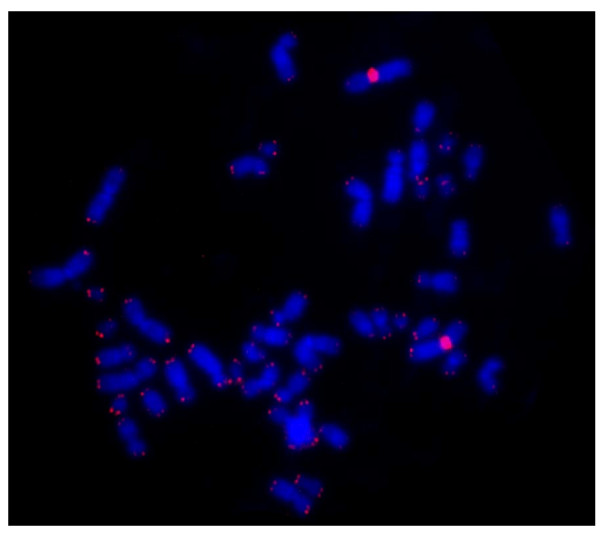
**U373 metaphase hybridized with telomere PNA probe labeled with Cy3**. Co-hybridisation was done with Cy-3 telomeric-PNA probe and Cy-3 centromeric-PNA(cromosome 2) probe.

To observe the correlation between astrocytic apoptosis and telomeres shortening the Q-FISH staining was performed on metaphase using the fluorescent PNA telomeric probe labeled with Cy3. Co-hybridisation was done with Cy-3 telomeric-PNA probe and Cy-3 centromeric-PNA(chromosome 2) probe. Each data was shown as percentage of T/C (telomere/centromere) ratio (Figure 3). At the same time point of apoptosis evaluation, the analysis of telomere length showed a statistically significant telomere shortening in HIV-exposed samples compared to negative control of mock HIV-1 exposed cells; this is in agreement to the observed increase of apoptotic cells (Figure 1 and 2) indicating a possible telomere role in cellular surviving, at the days of the analysis. On the other hand, the results obtained from NAC pretreatment of HIV-1 exposed cells at days 1, 3 and 5, show a statistically significant inhibition of telomere shortening indicating a telomere protective effect of this antioxidant against the oxidative stress-induced damage (Figure 3). In the figure 3 it is shown an interesting telomere lengthening, probably due to the NAC activity against an endogen oxidative intracellular status. In figure 4 we can observe a color image Q-FISH staining on metaphase performed on metaphase chromosomes with fluorescent PNA telomeric probe.

With these results we can suppose that the telomeres, preferential target for oxidative damage, could be the key sensor of cell apoptosis induced by oxidative stress after HIV-1 exposure.

### Telomerase activity is not modulated in HIV-1 exposed and/or NAC treated human astrocytoma cell line

**Figure 5:**
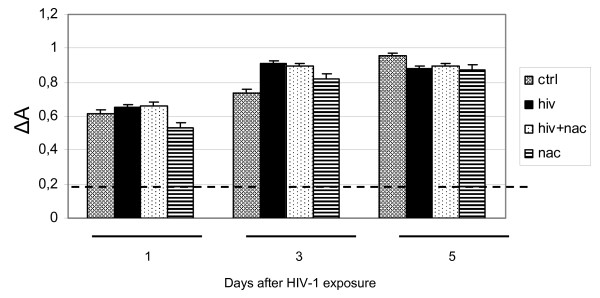
**Telomerase activity in U373 at different time points (1, 3, 5 days) following HIV-1 exposure and NAC treatment**. Data obtained on telomerase activity using the TRAP assay indicate no telomerase modulation in HIV- and NAC+HIV- treated U373 cells.

To evaluate if the reduction of telomere shortening is due to the telomerase modulation, we analyzed the telomerase activity in U373 exposed to HIV-1 by using the TRAP assay, at the same treatment time points used for Q-FISH analysis. As expected, U373 cell line express telomerase activity as usually observed in tumor cell lines. Interestingly, the data indicate no telomerase modulation in HIV- or NAC treated- HIV exposed U373 cells compared to the control. In fact, where we observed telomere shortening, we can't point out an enhanced or reduced telomerase activity (Figure 5). These data indicate no correlation between telomere length modulation and telomerase activity suggesting that NAC is able to act preventing the telomere shortening not by restoring the telomere length suggesting that the antioxidant protective effects does not occur via telomere elongation but most likely by restoring the oxidant status of the cells.

### HIV-1 modulates the GSH levels and GSH/GSSG ratio in human astrocytoma cell line

**Figure 6:**
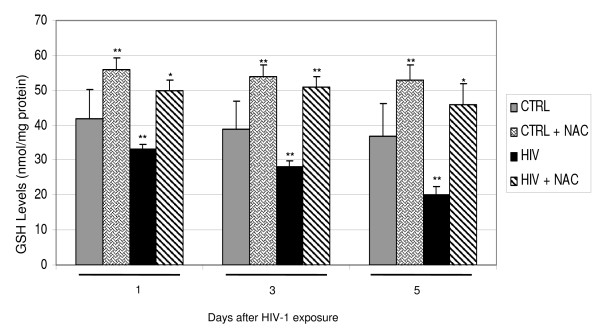
**Effects of NAC on GSH value**. In control experiments GSH level was expressed as nmol/mg protein and * p < 0.01; ** p < 0.05; *** p < 0.001 when compared with control; §p < 0.01; §§§ p < 0.001 when compared with HIV. F: 13.42, 18.60, 11.78, respectively at day 1, 3, 5. The data represent mean + SEM %.

HPLC was employed for evaluation of the cellular levels of the reduced or oxidized form of the cysteine-containing peptide glutathione (GSH and GSSG, respectively) in U373 exposed to HIV-1 IIIB and exposed, where requested, to NAC. As it is shown in the figure 6 the levels of GSH (nmol/mg protein), measured by HPLC, in U373 exposed to HIV-1 IIIB, decreased compared to negative control of not HIV-1 exposed cells at several days (day 1, day 3, day 5). Moreover we can observe that the NAC-treatment of U373 HIV-1 exposed, is able to restore the GSH levels at the same time points. The GSH/GSSG ratio, in U373 exposed to HIV-1 IIIB, also decreased compared with control of not HIV-1 exposed cells, the ratio significantly increased with NAC treatment (table 1).

**Table 1:** Effects of HIV-1 on GSH/GSSG ratio, a sensitive indicator of oxidative stress

GSH/GSSG (% Control)			
TIME (Day)	1	2	3

CTRL	100 ***	100**	100 ***
HIV	51 ± 6.2 ^§^	46 ± 6.3^§^	35 ± 2.8 ^§^
HIV + NAC	72 ± 4.3	68 ± 8.7	48 ± 5.1

The GSH/GSSG ratio, in U373 exposed to HIV-1 IIIB, decreases compared with control of not HIV-1 exposed cells. The ratio significantly increased with NAC treatment. *p < 0.05, **p < 0.01, ***p < 0.001 when compared vs control; §p < 0.05, §§ p < 0.01, §§§ p < 0.001, when compared vs HIV.

## Discussion

HIV infection is still pandemic with more than 30 million people infected today. In Europe, most of the 50% of HIV-affected patients and about 80% of AIDS affected patients undergo cognitive dysfunction which is indicated, at the late stage, as AIDS-dementia complex [32-34]. The neuropathogenesis of HIV-infection and therapeutic approaches for treatment of neurological disturbances accompanying AIDS still remain to be identified. Overall data suggest that the mechanism(s) that lead to neuronal, as well as non-neuronal damage, in the brain of AIDS patients may involve the combined effect of more than one neurotoxic factor. Oxidative stress and the alteration of the homeostasis induced by HIV-1 infection, have shown to contribute to the mechanisms underlying apoptotic cell death occurring in AIDS-dementia complex [1]. It is known that enhanced oxidative stress, which occurs in brain tissues of patients undergoing HIV infection and is implicated in apoptotic cell death of both astroglia and neurones, may play a role in the pathogenesis of neuroAIDS and it is also known that HIV-1-infected patients are under chronic oxidative stress [32,35-37]. Evidence exists that HIV infection is accompanied by simultaneous activation of free radical species in CNS cells other then superoxide anions, such as nitric oxide (NO) [1]. Apoptotic death of astrocytes could indirectly contribute to brain atrophy [38]. The design of this study was based on the crucial importance of oxidative stress induced by HIV-1 infection on the astrocytic damage. In particular, the aim of our research was to investigate the pathophysiological role of oxidative/inflammatory processes consequent to HIV-1 infection, in the development of apoptosis in the human astrocytic cell line U373. On the other hand, with the aim to correlate HIV-related apoptotic cell death with telomere dysfunction, we evaluated the telomere length changes and telomerase activity occurring in U373 exposed to HIV and undergoing oxidative stress subsequent to HIV-related inflammatory processes. In addition, the HIV-related generation of oxidative stress, has been correlated with the changes occurring on GSH/GSSH levels, involved in the regulation of endogenous antioxidants such as glutathione. GSH is the major thiol present in the brain tissue and the most important redox buffer in the cells [39]. This antioxidant molecule cycles between GSH and GSSG, and serves as a vital sink for control of ROS levels in cells. In our experiments we observed that HIV induced apoptosis is strictly related to changes occurring in GSH/GSSG ratio and GSH decrease suggesting the crucial role of oxidative stress in HIV related cellular damage. The presence of the antioxidant NAC was able to reduce the apoptosis occurring in astrocytoma cell line after 3 and 5 days of HIV virus exposure (71.1% and 47.1%, respectively Figure 1). This reduction of apoptosis can be considered more high if we take in account that the percentage of apoptosis in the mock-infected and NAC-untreated U373 at day 5 is around 17%. A confirming experiment of apoptosis was done by using the electron microscopy (Figure 2) and confirmed the results obtained with cytofluorimetric analysis. To observe if the astrocytic apoptosis is correlated to nuclear damage we analyzed the telomere shortening by Q-FISH staining. The telomere length in HIV-exposed samples was significantly shorter compared to control in keeping with the increase of apoptotic cells at the same time points, indicating a possible role of telomeres in the cellular surviving. NAC treatment shows a protective effect on HIV induced telomere shortening by inhibiting the oxidative stress-induced damage and restoring the GSH/GSSG ratio (Figure 3, Figure 6 and table 1). A statistically significant telomere lengthening was revealed at day 1 in NAC treated but not HIV-1 exposed cells, compared to negative control; this interesting telomere lengthening is probably due to the activity of the drug against an endogen oxidative intracellular status. It is in fact known that malignant cells produce height levels of oxidative stress to suppress apoptosis, accelerate proliferation, metastasis and angiogenesis [40]. For this reason in our cellular model we already have a basal level of oxidative compounds that can be removed by the NAC alone (a glutathione precursor).

High levels of telomerase activity have been demonstrated in immortalized cell lines [32] and in majority of human cancers [41,42] in which these cells that constitutively express telomerase can continue to divide almost indefinitely [19]; differently somatic cells, do not express telomerase, and their replicative potential are limited by progressive telomere shortening.

As expected, U373 cell line express telomerase activity as usually observed in tumor cell lines. Moreover, our results interestingly showed no statistically significant telomerase modulation in HIV and NAC+HIV- treated U373 cells (Figure 5) compared to the control, as observed by TRAP assay, despite telomere length modulation observed. This data could suggest two possible explanations for telomere lengthening observed in NAC pre-treated samples: the involvement of a telomerase-independent mechanism, the ALT pathway (Alternative Lengthening of Telomeres) [41,42]. In fact, it is well known that, consistent with the requirement for telomere maintenance as a step in carcinogenesis, 80–90% of human tumor possess telomerase activity and the remainder maintains telomeres via ALT, a recombination-mediated process [42]. On the other hand, this data could suggest that NAC is able to prevent the telomere shortening not by inducing the telomere lengthening, but most likely inhibiting the oxidative stress and restoring the cellular homeostasis as shown by the analysis of the levels of GSH and GSSG (ratio of reduced glutathione to oxidized glutathione) in HIV-1 exposed U373 and in NAC treated HIV-1 exposed U373. It is known that HIV infection is associated with decreases in the GSH content [43,44]. This decreased GSH content may reduce the survival of HIV-infected patients [45] perhaps by contributing to several disorders, such as CD4+ T cell apoptosis [46], neuroAIDS [47] and enhancing HIV replication. The figure 4 shows that the levels of GSH in U373 exposed to HIV-1 IIIB decreased compared to control; NAC treatment reversed this decrease and GSH returned to control levels. Moreover, as we can see in the table 1, the GSH/GSSG ratio at the days 1,3,5, in HIV-1 exposed U373 decreased compared to negative control of not HIV-1 exposed cells; at the same time points, the GSH/GSSG ratio significantly increased with NAC treatment. NAC-treatment of U373 HIV-1 exposed is able to restore the GSH levels, one of the major endogenous antioxidant molecule present in high levels in the cells. Several authors have suggested that low GSH levels were a consequence of decreased levels of plasma cysteine, the rate-limiting amino acid for GSH synthesis [34]. For this reason the presence of NAC, a cysteine donor involved in the synthesis of GSH, underlining that NAC can be a potential pro-GSH compound acting as part of the glutathione (GSH/GSSG) redox system.

## Conclusion

Our data demonstrated that, as shown in human fibroblast [23,27], in human astrocytic cell line U373, the increase of oxidative stress, consequent to HIV-1 exposure, is responsible of acceleration of telomere shortening "in vitro". So, we can suppose that the telomeres could represent a key sensor of cell apoptosis induced by oxidative stress following HIV-1 exposure. These observations can be an important starting point for future experiments. In addition, we demonstrated the protective action of antioxidant compound in reducing the HIV-1 IIIB-mediated cellular damage.

## Methods

### Cells

Glioblastoma cell line (U373) was obtained from the American Type Culture Collection (ATCC; Manassas, VA, USA) and grown, at 37°C and 5% CO2, in DMEM (Gibco, Grand Island, NY) supplemented with 10% heat-inactivated fetal bovine serum (FBS, Euroclone, Ltd, UK), Gentamicin 20 ug/ml, 2 mM L-glutamine.

### Virus

HIV-1 strain used in the preliminary experiment was R5-using Bal, and the HIV-1 strain used for the other experiments was X4-using IIIB. Both the viruses are provided by R. C. Gallo and M. Popovic at that time at the National Cancer Institute, National Institutes of Health, Bethesda, MD. The virus used for the experiments was ultra-centrifuged for two hours at 22,000 rpm at 4°C, stored in phosphate buffered saline (PBS) at -80°C.

### Analysis of cellular apoptosis

U373 were seeded in Petri plates (60,000 cells/plate, Costar, Cambridge, MA) and, 24 hrs later, were exposed, where requested, to 1 mM NAC for 20 min. Then, 8,000 pg/ml of p24-gag HIV-1 IIIB were added to the medium (3 ml of medium culture). The cells were then incubated at 37°C in humidified air containing 5% CO2. On the day of analysis, the cells were gently detached with trypsin/EDTA (0.02%) and centrifuged at 1,600 rpm for 10 min. Pellets were washed with PBS, placed in ice, and permeated with ice-cold 70% ethanol for 30 min. The aliquots were centrifuged at 1,500 rpm for 10 min, the pellets were washed with PBS, incubated with propidium iodide (PI; 100 μg/ml, SIGMA-Aldrich, Germany) and RNase (250 μg/ml Qiagen, Mi, Italy) at 4°C for 2 h in the dark. Samples were then washed twice with PBS and PI-stained cells were analysed by monitoring the incorporation of intracellular PI with a FACScan flow cytometer. Results are from 3 separate experiments; 10^5 ^events were collected for each sample. Data were acquired and analysed by the Lysis II program (Becton Dickinson, Buccinasco, Mi, Italy).

### Electron microscopy

Cells for electron microscopy (1 × 10^6^/ml) were fixed in 2.5% glutaraldehyde in PBS, pH 7.4, at 4°C and then washed for 2 times in PBS and post-fixed in osmium tetroxide, 1.33% for 2 h at 4°C. After several washes in PBS, the cells were dehydrated in graded alcohol, transferred into toluene, and embedded in Epon 812 resin. The resin was allowed to polymerize in a dry oven at 60°C for 24 h. Thin sections were cut and stained with toluidine blue, and examined on an Axioscope microscope. Ultrathin sections were cut on a Reichert microtome using a diamond knife, stained with uranyl acetate/lead-citrate, and evaluated and photographed on a Philips electron microscope CM 10 (Philips Electronic Instruments, Mt. Vernon, NY).

### Evaluation of telomeric length

cells metaphases, obtained treating samples for 2,5 hours with colchicine (10^-6 ^M), were treated with hypotonic KCl buffer (0.075 M) and successively fixed with methanol: acid acetic (3:1). Cells were therefore seeded onto glass slides and stored at RT for 48 hours. Metaphase chromosomes were analyzed by Quantitative-Fluorescent In Situ Hybridization (Q-FISH) with peptide nucleic acid (PNA)-telomeric probe. Briefly, after washing with Tris Buffered Saline (TBS), slides were fixed in formaldehyde (3.7%), treated with proteinase K, dehydrated through a series of ethanol rinses (70%–85%–100%) and air-dried. Probe mixture containing Cy-3-conjugated (C3TA2)3 peptide nucleic acid (PNA) and Cy-3 centromeric-PNA(chromosome 2) probe (DAKO, Glostrup, Denmark) was added to the slides, and a DNA/probe co-denaturation (3 min at 80°C) was carried out. After hybridization for 2 hours at room temperature, slides were washed in 4 × SSC + 0.01%Tween20 for 5 min at 65°C and dehydrated in an ethanol series (70%–85%–100% for 2 min). Finally, slides were counterstained with DAPI in Vectashield (Vector Laboratories, Burlingame, CA).

Metaphases were captured and karyotyped using dedicated software. Telomeres length of each chromosome was measured by ISIS/Telomere software (MetaSystems) which allows precise measurement of single telomere signal. Centromere 2 was used as reference signal allowing to calculate the Telomere/Centromere ratio (T/C ratio). Each data was shown as percentage of T/C ratio. Statistical analysis was calculated by comparing >1800 telomere values measured at least 10 metaphases for each experimental point.

### Estimation of telomerase activity

the PCR-based telomeric-repeat amplification protocol (TRAPeze ELISA Telomerase detection Kit) in accordance with the method proposed by Kim et al., [48] was used to evaluate the activity of telomerase. This procedure is separated in three steps: 1) Extract Preparation, proteic extract of untreated and HIV-treated cells was obtained by using 1× CHAPS lysis buffer (10 mM Tris HCl, 1 mM MgCl2, 1 mM EGTA, 0,1 mM PMSF, 5 mM β-Mercaptoethanol, 0,5% CHAPS, 10% glicerol) 2) TRAP extension/amplification, where telomerase present in proteic extract, adds a number of telomeric repeats (GGTTAG) onto the 3' end of a biotinylated substrate oligonucleotide (b-TS); the extended products are then amplified by PCR. 3) Detection (ELISA). PCR products were analysed and the absorbance (A) was evaluated by the microplate reader. The difference between the absorbance for the sample and heat-treated sample was indicated as ΔA. When ΔA > 0.150 the sample is defined telomerase positive.

### Measurement of GSH and GSSG

we measured the oxidative stress analysing the GSH value and the GSH/GSSG ratio, as is already well documented by the literature [49-51]. Specifically, intracellular cell GSH and GSSG content were determined by High-Performance Liquid Chromatography (HPLC) according to Reed et al. [52]. Briefly, U373 cells were gently scraped off, washed and harvested by centrifugation at 2,000 rpm in a refrigerated centrifuge. Cell samples were suspended in phosphate-buffered saline and then lysed by repeated cycles of freezing and thawing under liquid nitrogen. Proteins were precipitated by adding sodium metaphosphoric acid (MPA) to a final concentration of 5% (w/v). A 0.5 ml aliquot of the clear supernatant, was treated immediately with 50 ul of a fresh aqueous solution (4 umol) iodoacetic acid and then neutralized with an excess of NaHCO3 (dry powder). After 60 min in the dark at room temperature, 0.5 ml of an alcoholic solution of 1-fluoro-2,4-dinitrobenzene (1.5 ml/98.5 ml absolute ethanol) was added and allowed to react overnight in the dark [52]. Aliquots (100 ul) of the reaction mixtures were subjected to HPLC analysis. Protein levels of the cell samples were determined by the Bredford method [52,53].

### Trypan blue-exclusion test of cell viability

The dye-exclusion test was used to determine the number of viable cells after exposure of astrocytes to NAC. At days 1, 3 and 5, astrocytes were trypsinized, exposed to dye, and then examinated visually to determine whether cells take up or exclude dye. The live cells that possess intact cell membranes exclude trypan blue, whereas dead cells do not [54].

### Statistical analysis

All the results were given as mean ± sem. Data are from 3 separate experiments, each experiment was run in triplicate. These results were performed using ANOVA followed by Student-Newman-Keuls unless specified (n = 3 different experiments). P < 0.05 was considered statistically significant.

## Authors' contributions

All authors contributed to the design of the experiment. MP contributed to all the experiments, performed the cytofluorimetric analysis and drafted the manuscript. CM and AS contributed to the data analysis and the draft of the manuscript. AB contributed the Q-FISH staining and TRAP assay. VM participated in the design of the study and in its coordination. CT participate in the design of the study. CDD and PR contributed to the GSH and GSSG evaluation and to the data analysis. CFP and SA conceived of the study, participated in the design of the study and in its coordination. All authors contributed to manuscript preparation, and approved the final manuscript.
